# Experimental and theoretical investigations of the effect of bis-phenylurea-based aliphatic amine derivative as an efficient green corrosion inhibitor for carbon steel in HCl solution

**DOI:** 10.1016/j.heliyon.2023.e20254

**Published:** 2023-09-22

**Authors:** Mohanad Shkoor, Rem Jalab, Mazen Khaled, Tahseen S. Shawkat, Hesham M. Korashy, Mohamed Saad, Haw-Lih Su, Abdulilah Dawoud Bani-Yaseen

**Affiliations:** a Department of Chemistry & Earth Sciences, College of Arts & Science, Qatar University, P.O. Box 2713, Doha, Qatar; b Gas Processing Center, College of Engineering, Qatar University, P.O. Box 2713, Doha, Qatar; c Department of Pharmaceutical Sciences, College of Pharmacy, Qatar University, P.O. Box 2713, Doha, Qatar; d Department of Chemical Engineering, College of Engineering, Qatar University, P.O. Box 2713, Doha, Qatar; e Department of Applied Chemistry, National Chiayi University, 300 Syuefu Rd, Chiayi City, Taiwan

**Keywords:** Green corrosion inhibitor, Bis-phenylurea aliphatic amine, Carbon steel, Adsorption isotherm, Electrochemical measurements, Density functional theory

## Abstract

A novel bis-phenylurea-based aliphatic amine (BPUA) was prepared via a facile synthetic route, and evaluated as a potential green organic corrosion inhibitor for carbon steel in 1.0 M HCl solutions. NMR spectroscopy experiments confirmed the preparation of the targeted structure. The corrosion inhibitory behavior of the prospective green compound was explored experimentally by electrochemical methods and theoretically by DFT-based quantum chemical calculations. Obtained results revealed an outstanding performance of BPUA, with efficiency of 95.1% at the inhibitor concentration of 50 mg L^−1^ at 25 °C. The novel compound has improved the steel resistivity and noticeably reduced the corrosion rate from 33 to 1.7 mils per year. Furthermore, the adsorption study elucidates that the mechanism of the corrosion inhibition activity obeys Langmuir isotherm with mixed physisorption/chemisorption modes for BPUA derivatives on the steel surface. Calculated Gibb's free energy of the adsorption process ranges from −35 to −37 kJ mol^−1^.

The SEM morphology analysis validates the electrochemical measurements and substantiates the corrosion-inhibiting properties of BPUA. Additionally, the eco-toxicity assessment on human epithelial MCF-10A cells proved the environmental friendliness of the BPUA derivatives. Density functional theory (DFT) calculations correlated the inhibitor's chemical structure with the corresponding inhibitory behavior. Quantum descriptors disclosed the potentiality of BPUA adsorption onto the surface through the heteroatom-based functional groups and aromatic rings.

## 1 Introduction

Corrosion is a common challenge in many crucial industries, especially petroleum, petrochemical, and desalination [[1], [2], [3], [4]]. These industries frequently operate under extreme acidity or salinity conditions, requiring extensive investigation of the corrosion chemistry. Corrosion is essential in oil and gas exploration activities, especially when attacking transportation pipelines. The commonly applied practices of wells acidifications, acid descaling, and pickling for scrubbing metals and alloys result in the loss of metallic materials [5]. For instance, cleaning processes are usually conducted using 1.0 M HCl acid, whereas acidizing treatments are performed with (15–28 wt%) HCl [6,7].

Among the most important chemicals used in the fields are corrosion inhibitors to protect metallic equipment from corrosion, specifically carbon steel. However, there is a growing concern from governments and environmental agencies regarding these chemicals' toxicity and consequent ecological impact. A typical corrosion inhibitor needs to provide appropriate protection for the most widely used carbon steel against acidity since acids, especially hydrochloric acid, are utilized in many processes, such as in fracking, removal of carbonates reservoirs, and chemical cleaning. This requires developing effective techniques to mitigate and slow down the corrosion of metals, especially steel, which is the primary metallic material that suffers from acid attack and degradation [8].

Corrosion inhibitors have been utilized for decades; however, there is pressure on the gas and oil industry to shift towards more environmentally benign and low-toxicity corrosion inhibitors. Interestingly, growing interest has recently been noted in utilizing organic corrosion inhibitors for industrial processes due to their environmental friendliness and wide temperature range [[9], [10], [11], [12], [13], [14], [15], [16], [17]]. Ideally, such corrosion inhibitors must be obtained from low-cost materials that are environmentally friendly and selected from compounds incorporating heteroatoms, aromatics, or long carbon chains [18,19]. Hence, such functional groups can facilitate the adsorption of the corrosion inhibitor on the metal surface and consequently promote the inhibition process.

Urea, which is an organic compound with two amine groups in the structural formula, has been the focus of many researchers by utilizing its electron-rich heteroatoms to design and synthesize several corrosion inhibition structures [7]. Urea-derived molecules displayed enhanced corrosion inhibition properties for steel in aggressively corrosive media, including HCl, H_2_SO_4_, and NaCl [20]. In the work of Padmashree et al. [21], 1,3-bis(1-phenylethyl) urea demonstrated a promising performance for corrosion inhibition of carbon steel in 1.0 M HCl, reaching around 72% efficiency at 305 K and 80 mg L^−1^ concentration. Additionally, 1-phenyl-2-thiourea (PTU) and 1,3-diisopropyl-2-thiourea (ITU) compounds were studied electrochemically for mild steel in 1.0 M HCl [22]. The study revealed the potential of 0.005 M PTU in achieving around 99% inhibition efficiency at 60 °C. It is extensively reported that several urea and thiourea derivatives are employed as corrosion inhibitors for the pickling processes utilizing solutions based on HCl, H_2_SO_4_, HNO_3_, and H_3_PO_4_ [[23], [24], [25]]. The abundance of lone pair electrons in their structures creates active sites for interacting with the steel surface, enhancing adsorption potential.

In our current work, the synthesis of a novel bis-phenylurea-based aliphatic amine compound (BPUA), namely 1,1'-((methylazanediyl)bis(propane-3,1-diyl))bis(3-(*p*-tolyl)urea), as a green corrosion inhibitor for carbon steel in 1.0 M HCl solution is reported for the first time. Since practices for oil wells acid pickling and descaling treatments involve the injection of 1.0 M HCl solution, it is of significant importance to study the corrosion behavior under the conditions confronting the gas and oil industry. The performance of the developed BPUA compound is evaluated experimentally in the acidic medium by employing electrochemical techniques of electrochemical impedance spectroscopy (EIS) and potentiodynamic polarization (PDP). The scanning electron microscope (SEM) was used to determine the morphology and surface corrosion properties before and after corrosion. Despite this, the environment-friendly characteristics of BPUA are evaluated experimentally using epithelial cells (MCF-10) and theoretically via ADMET models web tools. Density Functional Theory (DFT)-based chemical quantum calculations were conducted to correlate the inhibitor's chemical structure with the corresponding inhibitory behavior.

## 2 Experimental

### 2.1 Materials

Fine chemicals and solvents were purchased from Sigma-Aldrich and were utilized without additional purification. All organic solvents used in spectroscopic studies were of spectroscopic grades and used as received.

### 2.2 Synthesis of BPUA; 1,1'-((methylazanediyl)bis(propane-3,1-diyl))bis(3-(*p*-tolyl)urea)

**Scheme 1:**
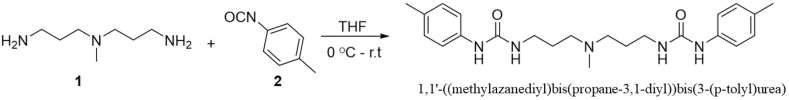
Synthetic route of the bis-urea based inhibitor (BPUA).

A solution of 3,3′-diamino-N-methyldipropylamine **1** (1.5 g, 10 mmol, 1 eq.) in THF (15 ml) was added dropwise to a cold solution of *p*-tolyl isocyanate **2** (2.6 g, 20 mmol, 2 eq.) in THF (15 ml). After the addition was completed, the temperature was allowed to rise gradually to room temperature, upon which a white precipitate began to form. The mixture was stirred for 4 h to ensure a complete reaction. The formed precipitate was collected by vacuum filtration and later purified by crystallization from a hot THF to give white crystals of m.p. 196–198 °C. The elemental analysis of the compound was in agreement with the calculated analysis, as the calculated analysis was C, 67.13; H, 8.08; N, 17.02, and the found elemental analysis was C, 67.54; H, 8.11; N, 17.21. The structure of the prepared compound (BPUA derivative) was confirmed by spectroscopic methods (supplementary materials). The synthetic route of BPUA inhibitor is displayed in Scheme 1.

### 2.3 Electrochemical measurements

A plate corrosion cell was used with a carbon steel (C-steel) coupon with 1.0 cm^2^ exposed to the HCl solution representing the working electrode. A graphite rod was used as a counter electrode, and a silver/silver chloride electrode was utilized as a reference electrode. The potentiostat used to carry out the potentiodynamic and AC impedance experiments was made by Gamry. The Gamry fitting software used to fit the electrochemical curves were EIS300 and DC105, respectively. C-steel corrosion was investigated against 1.0 M HCl corrosive medium in the absence and presence of different concentrations of BPUA inhibitor (10, 20, 30, and 50 mg L^−1^) at 25 °C. The coupon was immersed in the solution for a duration of 1 h prior to commencing the corrosion tests in order to establish a stable free corrosion potential. Electrochemical impedance spectroscopy (EIS) was performed within a frequency range of 100 mHz to 1 × 10^2^ kHz, with 10 mV AC amplitude. The potentiodynamic polarization (PDP) experiments were carried out in a potential range of −250 and + 250 mV with respect to the open circuit potential vs Ag/AgCl at a scan rate of 0.166 mV s^−1^.

### 2.4 Surface characterization

Scanning electron microscopy (SEM) was performed to determine the effect of the corrosion inhibitor concentrations on the surface properties of the steel specimen before and after corrosion. Immersion tests for 12 h were conducted in 1.0 M HCl to evaluate the effect of the corrosive hydrogen chloride. Three steel coupons were polished to a mirror-like finish. Polished samples were immersed in 50 mg L^−1^ BPUA-inhibited and uninhibited 1.0 M HCl solutions. The SEM used was a NovaNano SEM450 (ThermoFisher, Netherlands) connected with Energy Dispersive X-Ray (Bruker, Germany).

### 2.5 Eco-toxicity assessment

#### 2.5.1 Experimental approach from cell viability

The impact of the produced chemical on the survival of MCF-10 normal epithelial cells was assessed. The MCF-10A human epithelial breast cells were obtained from the American Type Culture Collection (Rockville, MD). These cells were maintained in Dulbecco's Modified Eagle Medium (DMEM) with phenol red, supplemented with 10% fetal bovine serum and 1% 100× Antibiotic-Antimycotic. The cells were cultured in 75-cm^2^ tissue culture flasks and kept in a humidified environment with 5% CO_2_ at a temperature of 37 °C. A novel stock solution of BPUA was created using Dimethyl Sulfoxide (DMSO) as the solvent, ensuring that the concentration of DMSO did not surpass 0.25%.

The viability test assesses the enzymatic capability of the living cell's reducing enzyme to convert [4,5-dimethyl-thiazol-2-yl]-2,5-diphenyltetrazolium bromide (MTT) into formazan crystals with a distinct coloration. The MCF-10A cells were subjected to treatment with different doses of the test chemical on a 96-well plate (Corning Incorporated, USA) for a duration of 24 h. This experiment was conducted at a temperature of 37 °C inside a 4% CO_2_ humidified incubator. Subsequently, the cell culture medium was aspirated, and the cells were exposed to 100 μL of MTT solution (0.5 mg ml^−1^ in phosphate-buffered saline) per well. This incubation was carried out for a duration of 3 h in a carbon dioxide incubator set at a temperature of 37 °C, while ensuring protection from light. Next, the medium was separated from the plate by inverting it, and afterwards, 100 μL of isopropyl alcohol was introduced into each well to dissolve the formazan crystals. The plate was then shaken for a duration of 5 min. The absorbance at a wavelength of 570 nm was determined using a Multiskan SkyHigh spectrophotometer, which is an automated microplate reader. The calculation of cell viability percentage was performed by comparing it to the control wells, which were defined as having 100% viable cells.

#### 2.5.2 Theoretical approach from ADMET models

ADMETSAR web tool [26,27] was utilized to assess the eco-toxicity of the synthesized BPUA inhibitor. This tool employs a machine-learning model formulated from 210,000 experimental entries of 100,000 compounds to evaluate the absorption, distribution, metabolism, excretion, and toxicity properties. The water solubility was predicted from the SwissADME website [28,29], which also uses a model based on 2874 solubility measurements against nine properties [30].

### 2.6 Computational methods

The DFT calculations were conducted using Gaussian 09 version D.01. The molecular geometry optimization was performed using the hybrid B3LYP functional and the 6-31 + G(d) basis set [31]. The integral equation formalism polarizable continuum model (IEFPCM) was used to account for the implicit solvent effect [32]. The optimized geometry was confirmed as a minimum in the potential energy surface with no imaginary frequency. The geometry of the inhibitor was optimized for the neutral (N), cationic (N+1), and anionic (N-1)forms in the implicit aqueous medium. All quantum and energy parameters of the inhibitor were calculated at the same level of DFT theory using the equations below; this includes the highest occupied molecular orbital (E_HOMO_), the lowest unoccupied molecular orbital (E_LUMO_), the energy gap (ΔE) (eq (1)), the ionization potential (I) (eq (2)), electron affinity (A) (eq (3)), chemical electronegativity (χ) (eq (4)), absolute hardness (η) (eq (5)), and fraction of electrons transferred (ΔN) (eq (6)) between the inhibitor and metal surface:

(1) $$\Delta E=E_{\text{LUMO}}-E_{\text{HOMO}}$$

(2) $$I=-E_{\text{HOMO}}$$

(3) $$A=-E_{\text{LUMO}}$$

(4) $$\chi =\frac{I+A}{2}$$

(5) $$\eta =\frac{I-A}{2}$$

(6) $$\Delta N=\frac{\chi_{Fe}-\chi_{Inh}}{2\left(\eta_{Fe}+\eta_{Inh}\right)}$$

The local reactivity of the inhibitor was calculated on the basis of the Fukui reactivity indices (eq (7) and eq (8)) utilizing the Muliken atomic charge density (q_k_) of the optimized geometry of the neutral, cationic, and anionic forms of the inhibitor per the equations below:

(7) $${ƒ}_{k}^{+}=q_{k}\left(N+1\right)-q_{k}\left(N\right)$$

(8) $${ƒ}_{k}^{-}=q_{k}\left(N\right)-q_{k}\left(N-1\right)$$

## 3 Results and discussion

### 3.1 Synthesis and spectral characterization

The chemical structure of the synthesized bis-urea inhibitor BPUA was fully characterized by employing various NMR techniques. ^1^HNMR (Figure S-1, SI) and ^13^CNMR (Figure S-2, SI) experiments were performed to elucidate the structure of the targeted inhibitor. Additionally, 2D experiments were performed to confirm the proposed structure of BPUA. Heteronuclear Multiple Quantum Coherence (HMQC) is a heteronuclear 2D experiment showing cross-peaks that reveal the direct (^1^J) correlation between ^1^H and heteronuclei. At the same time, the Heteronuclear Multiple Bond Correlation (HMBC) gives correlations between heteronuclei and protons that are separated by two, three, and, sometimes in conjugated systems, four bonds. Moreover, the ^1^H–^15^N HMQC NMR spectrum of BPUA (Figure S-3, SI) indicates that ^1^H NMR signals at 6.14 and 8.28 ppm have cross-peaks with ^15^N NMR signals at 81 and 99 ppm, respectively, suggesting they are amide N–H's. The ^1^H–^15^N HMBC NMR spectrum (Figure S-4, SI) indicates that ^15^N NMR the signal at 99 ppm also couples to aromatic proton at 7.22 ppm while the ^15^N NMR signal at 81 ppm couples to ^1^H signals at 1.56 ppm and 3.09 ppm. In the ^1^H–^15^N HMBC NMR spectrum, the third ^15^N signal at 26.5 ppm is observed, suggesting a tertiary amine coupling to ^1^H signals at 1.56 ppm and 2.12 ppm. The ^1^H–^1^H COSY of the NMR spectrum of BPUA (Figure S-5, SI) shows strong couplings of aromatic protons between 6.99 and 7.22 ppm and of CH_2_–CH_2_–CH_2_ among 1.56, 2.30, and 3.09 ppm. In addition, two weak couplings were observed: the coupling between 6.99 ppm and 2.20 ppm suggests 2.20 ppm is the methyl group on tolyl; the coupling between 6.14 ppm and 3.09 ppm means the NH–CH_2_ structure. To this end, based on these characterization methods, the structure of the target BPUA is confirmed.

### 3.2 Corrosion and electrochemical behavior

#### 3.2.1 EIS measurements

**Fig. 1:**
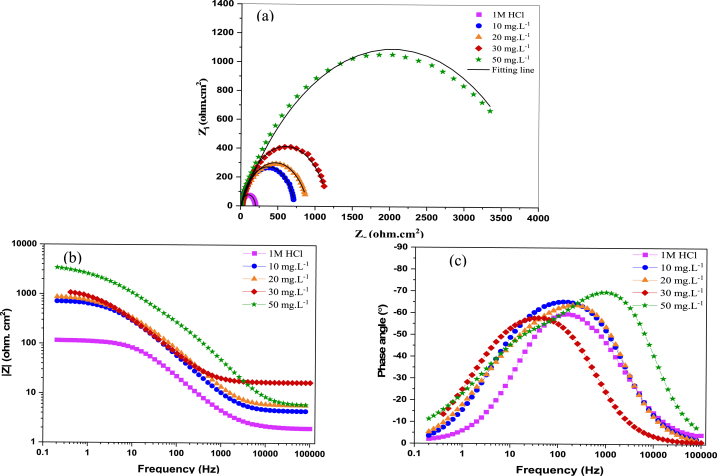
Effect of BPUA concentration on Nyquist (a), Bode magnitude (b), and phase angle (c) plots for C-steel in 1.0 M HCl at 25 °C.

The impedance spectrum of BPUA was acquired by the use of electrochemical impedance spectroscopy (EIS) measurements in order to investigate the impact of including inhibitors on the corrosion processes [33]. The use of EIS semicircles has provided valuable assistance in comprehending the interface between a surface and a solution, as well as the formation of an electric double layer. The rate of electron transport between cathodic and anodic sites is influenced by the formation of a double layer resulting from the accumulation of species on the metallic surface [34]. EIS curves comprising Nyquist, two forms of Bode plots: impedance modulus, and phase angle at 25 °C are shown in Fig. 1.

The augment of BPUA concentration is reflected in the expanded diameter of the EIS semicircles (Fig. 1 (a)). This suggests that the increased capacitive loop is ascribed to enhanced adhesion of BPUA molecules onto the C-steel interface, thereby increasing the charge transfer resistance [35]. The semicircles at concentrations of 10, 20, and 30 mg L^−1^ display a relatively close corrosion inhibition resistance for BPUA. However, the diameter of the semicircle is remarkably increased upon adding 50 mg L^−1^ of BPUA compared to that of the blank curve. This improved resistance trend proves the increased corrosion inhibition efficiency of BPUA in the 1.0 M HCl medium. The single semicircle in all Nyquist curves indicates a charge transfer-controlled C-steel disintegration. However, the incomplete semicircular Nyquist plots may be ascribed to the surface heterogeneity [36].

Furthermore, the two Bode plots demonstrate improved conditions at higher BPUA concentrations, as in Fig. 1 (b & c). It is evident that Bode plots show a trend with higher and broader curves upon the increase in inhibitor concentration. The low-frequency area in the impedance modulus plot (Fig. 1 b) possesses a noticeably boosted absolute modulus |Z| for the inhibited conditions. Therefore, this elucidates an outstanding inhibitory efficacy of BPUA, where the molecules work to block the active sites by adsorption onto the C-steel surface [36]. Nevertheless, the one-time constant appearing in the phase angle plots indicates the occurrence of the relaxation process during the inhibitor adsorption. The phase angle increased to 70° at 50 mg L^−1^ concentration. The negative shift in phase angle demonstrates the formation of a protective coating on the steel surface [37]. Overall, EIS outcomes confirm that BPUA creates a barrier film that can restrain the C-steel and retard the diffusion of corrosion species [38].

**Fig. 2:**
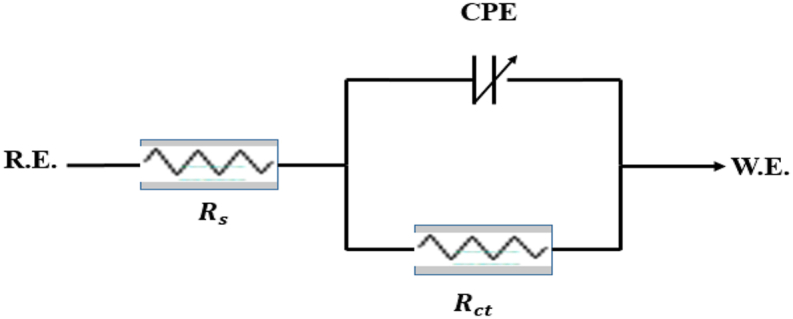
The equivalent circuit model for EIS fitting.

Typically, EIS data is analyzed by using an analogous circuit model that incorporates a single time constant. The circuit model presented below allows for the analysis of the impedance exhibited by an electrode undergoing uniform corrosion. (Fig. 2). The behavior of resulting Nyquist plots (Fig. 1) showing a single semicircle with no change in the expected impedance curves and the minor errors in the goodness of fitting are the main reasons for emphasizing the relevance of the selected fitting circuit. The circuit is composed of two main components, namely the solution resistance (R_s_) and the constant phase element (CPE), which are connected in parallel with the charge transfer resistance (R_ct_).

The capacitance of the double layer $\left(C_{\text{dl}}\right)$ is found from the charge transfer resistance and surface inhomogeneity (n) (eq (9)):

(9) $$\begin{array}{c} C_{\text{dl}}=\frac{{\left(Y_{0}R_{\text{ct}}\right)}^{1/n}}{R_{\text{ct}}} \end{array}$$

The thickness of the double layer is related to the capacitance via the below expression (eq (10)):

(10) $$\begin{array}{c} C_{\text{dl}}=\frac{\varepsilon \;\varepsilon_{0}A}{\delta } \end{array}$$

where $\varepsilon_{0}$ and ε are the dielectric constants for air and water, respectively. A is the cross-sectional area of the electrode.

Then, inhibition efficiency (IE%) can be determined from $R_{\text{ct}}$ values according to eq (11):

(11) $$\begin{array}{c} \text{IE}\%=\frac{R_{\text{ct}1}-R_{\text{ct}2}}{R_{\text{ct}1}}\times 100 \end{array}$$

the variables R_ct1 and R_ct2 represent the charge transfer resistance when the inhibitor is present and absent, respectively.

Fitted EIS parameters are summarized in Table 1, with outcomes asserting an increase in the charge transfer resistance, thereby elevating the inhibition efficiency. It is evident that adding 50 mg L^−1^ BPUA has a significant effect on boosting the charge transfer resistance ($R_{\text{CT}})$ by approximately 21 times. The novel structure of BPUA has noticeably increased $R_{\text{CT}}$ from 196 Ω cm^2^ for the uninhibited 1.0 M HCl to 4040 Ω cm^2^ at 50 mg L^−1^ concentration, achieving 95% efficiency. It is worth mentioning that BPUA is effective at as low as 10 mg L^−1^ concentration, yielding 73% corrosion suppression efficiency. Furthermore, the double-layer capacitance was at its minimum of 27.8 μF at the optimum tested concentration; the decline in $C_{\text{dl}}$ indicates an increase in the thickness of the electrical double layer, as illustrated in equation (10). In contrast, in uninhibited conditions without a resistive barrier, the C-steel usually exhibits severe corrosion corresponding to larger values of $C_{\text{dl}}$ [39].

**Table 1:** EIS parameters of C-steel corrosion in 1.0 M HCl in the absence and presence of different BPUA concentrations at 25 °C.

C_Inh._ (mg.L^−1^)	R_CT_ (Ω. cm^2^)	R_S_ (Ω. cm^2^)	CPE				IE%
			Y_0_ (μs^n^. Ω^−1^. cm^−2^)	n	Goodness of fit	$C_{\text{dl}}$ (μF)	
0	196	5.3	99.9	0.85	0.6E-04	49.9	**-**
10	727	4.2	92.6	0.82	0.8E-03	50.6	73.0
20	906	5.3	125	0.75	1.2E-02	59.2	78.4
30	1171	16.2	116	0.78	0.7E-03	66.8	83.3
50	4040	5.4	62.3	0.63	0.3	27.8	95.1

**Fig. 3:**
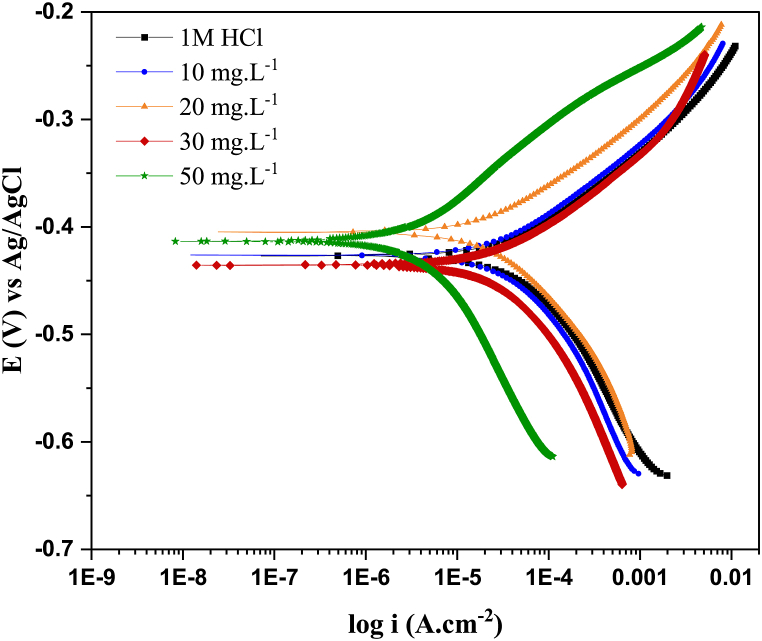
Effect of BPUA concentration on potentiodynamic polarization plots for C-steel in 1.0 M HCl at 25 °C.

*3.2.2 PDP.* Potentiodynamic polarization tests of C-steel in 1.0 M HCl in the absence and presence of BPUA inhibitor were performed at 25 °C, as shown in Fig. 3. Obviously, the anodic and cathodic branches in the plots of inhibited solutions with different BPUA concentrations shift to more positive and negative potentials, respectively. Consequently, the anodic and cathodic current densities notably declined after adding the BPUA inhibitor. This suggests that the structure of BPUA facilitates the attachment of molecules to the C-steel surface through free electron pairs around the aromatic moieties or heteroatoms [40]. The coordination bonds formed with BPUA and the steel surface obstruct the available active sites, thereby decelerating both reactions of the anodic metallic dissolution and hydrogen evolution [41]. Indeed, the shape of the polarization curves displays a similar trend after the adsorption of BPUA at the interface with the steel electrode, revealing a preserved reactive mechanism [35]. Corrosion parameters, including free potential ($E_{\text{corr}}$), current density ($i_{\text{corr}}$), polarization resistance ($R_{p}$), corrosion rate (CR), anodic ($\beta_{a}$) and cathodic ($\beta_{c}$) slopes of Tafel branches are given in Table 2, as acquired by the Tafel extrapolation method.

**Table 2:** Potentiodynamic polarization parameters of C-steel corrosion in 1.0 M HCl in the absence and presence of different BPUA concentrations at 25 °C using the Tafel extrapolation method.

C_Inh._ (mg.L^−1^)	-E_corr_ (mV)	i_corr_ (μA. cm^−2^)	$\beta_{a}$ (mV/decade)	$\beta_{c}$ (mV/decade)	$R_{p}$ (Ω. cm^2^)	CR (mpy)	IE%
Blank	420	76.6	53.7	99	197	33.6	–
10	−426	35.5	77.6	141	612	16.2	53.6
20	−405	27.5	73.6	124	729	12.6	64.1
30	−436	19.2	58.3	109	859	8.8	74.9
50	−413	4.0	76.0	143	5351	1.7	95.1

IE% is determined from the current density ($i_{\text{corr}}$) according to eq (12):

(12) $$\begin{array}{c} \text{IE}\%=\frac{i_{\text{corr}1}-i_{\text{corr}2}}{i_{\text{corr}1}}\times 100 \end{array}$$

where $i_{\text{corr}1}$ and $i_{\text{corr}2}$ are the corrosion current densities in the absence and presence of the inhibitor, respectively.

The polarization resistance ($R_{p})$ is found from the Stern–Geary equation (eq (13)):

(13) $$\begin{array}{c} R_{p}=\frac{\beta_{a}\beta_{c}}{2.303\;i_{\text{corr}}\left(\beta_{a}+\beta_{c}\right)} \end{array}$$

The listed parameters classify BPUA as a mixed-type inhibitor since the potential shift between uninhibited and inhibited conditions is less than 85 mV [42,43]. Additionally, the obtained data assert the impact of BPUA on declining the cathodic and anodic current due to the weakened Cl^−^ anions adsorption into the C-steel surface, where the occurring electrochemical processes are altered [44]. Nevertheless, the change in the slopes $\beta_{a}$ and $\beta_{c}$ as well as the E_corr_ is not very significant. This suggests that BPUA molecules are first adsorbed on the surface, then assist in a one-time reaction stopping on the restricted surface without varying the redox mechanism [45]. It can be considerably remarked that the polarization resistance is reinforced at higher BPUA concentrations, thereby enhancing corrosion resistance and reducing corrosion rates [46]. This indicates boosted electron density over the C-steel surface, ascribed to the accumulation of BPUA molecules forming a protective layer [47]. At the highest tested concentration of 50 mg L^−1^, Rp reached 5351 Ω cm^2^ compared to 197 Ω cm^2^ reported for the blank solution. The substantial reduction in the current density from 76.6 to 4.0 μA. cm^−2^ reveals the potential of BPUA to achieve 95% efficiency in diminishing the severity of corrosion. Consequently, this is essentially interpreted by the disclosed corrosion rate declining to 1.7 mils per year at 50 mg L^−1^.

### 3.3 Adsorption isotherm

**Fig. 4:**
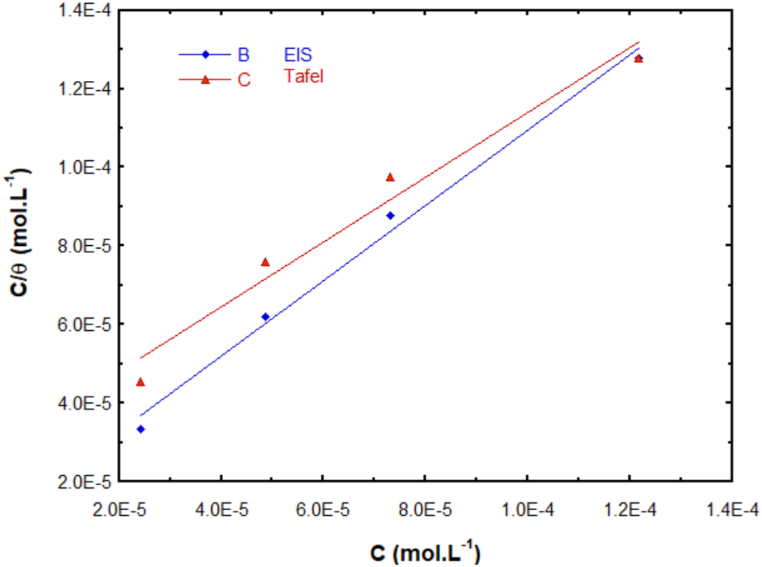
Langmuir adsorption isotherm BPUA on C-steel in 1.0 M HCl at 25 °C using the Tafel extrapolation and EIS methods.

The EIS and PDP experiments showed that inhibitory behavior of BPUA is an adsorption-controlled process, where BPUA adsorption on the surface of C-steel is critical for providing efficient inhibition. Intrinsically, the adsorption isotherms can provide essential information regarding the effectiveness of organic corrosion inhibitors in preventing corrosion. Within this context, selecting adsorption isotherm models entails fitting the degree of surface coverage (θ) obtained from the electrochemical data to various isotherms; this includes the Langmuir, Temkin, and Frumkin isotherms. The linear regression parameter (R) for each of the tested isotherms demonstrated that the adsorption of BPUA on the surface of C-steel follows the Langmuir isotherm according to eq (14):

(14) $$\frac{C}{\theta }=C+\frac{1}{K_{ads}}$$

where C is the inhibitor concentration, θ is the degree of surface coverage, and K_ads_ is the adsorption equilibrium constant. Fig. 4 shows the Langmuir isotherm constructed from fitting the corrosion experiments obtained by two methods, namely the Tafel EIS methods. As can be noted, the appropriateness of employing the Langmuir isotherm for describing the adsorption process of BPUA on the surface of C-steel is confirmed by the linear regression parameter, where values of 0.996 and 0.985 were calculated for the EIS and Tafel methods, respectively. The linear regression equation is employed to estimate the value of K_ads_ for each technique. Accordingly, the standard change in Gibb's free energy of the adsorption process (${\Delta G}_{ads}^{{}^{\circ}}$) can be calculated employing eq (15):

(15) $${\Delta G}_{ads}^{{}^{\circ}}=-RTln\left(55.5K_{ads}\right)$$

where T is the absolute temperature in Kelvin, and R is the universal gas constant. The obtained results are summarized in Table 3. These results revealed ${\Delta G}_{ads}^{{}^{\circ}}$ values of −37.8 and −35.6 kJ mol^−1^ for the EIS and Tafel methods, respectively. The high K_ads_ value and negative sign of ${\Delta G}_{ads}^{{}^{\circ}}$ suggest that the BPUA molecules are strongly adsorbed on the surface of C-steel via a spontaneous process, and consequent grander inhibitory ability can be predicted. Additionally, in the corrosion inhibition literature ΔG values ≤ −20 kJ/mol refers to the physisorption process, whereas when ΔG ≥ −40 kJ/mol indicates a chemisorption [48,49]. The calculated values of ${\Delta G}_{ads}^{{}^{\circ}}$ are indicative of a physisorption process for BPUA on the surface of C-steel; however, these values are close to the threshold of chemisorption, especially the value from EIS data, which in turn suggests that the adsorption process may proceed via mixed chemisorption and physisorption modes [10,[50], [51], [52]]. Notably, such adsorption behavior is a typical adsorption mode for an organic inhibitor. In addition to electrostatic interaction that can induce chemisorption, in principle, an organic molecule bearing heteroatoms-based functional groups as well as π-bonding electrons are capable of interacting with the surface of the metal via electrons transferred between the organic inhibitor and metal surface as well as electrostatic interaction that can induce chemisorption. As such, by examining the chemical structure of BPUA, one can notice that it bears heteroatom-based functional groups, namely amines and carbonyls, as well as aromatic rings, which indeed can facilitate the adsorption of BPUA molecules on the surface of C-steel via mixed adsorption modes.

**Table 3:** Adsorption parameters of Langmuir isotherm of BPUA on C-steel in 1.0 M HCl at 25 °C using the Tafel extrapolation and EIS methods.

Experimental method	Langmuir Isotherm			
	linear equation	R^2^	K_ads_ (L.mol^−1^)	${\Delta G}_{ads}^{{}^{\circ}}\;\left(kJ.{mol}^{-1}\right)$
EIS	y = 0.962x + 1.335E-5	0.992	7.49 × 10^4^	−37.8
Tafel	y = 0.826x + 3.142E-5	0.971	3.18 × 10^4^	−35.6

### 3.4 SEM characterization

**Fig. 5:**
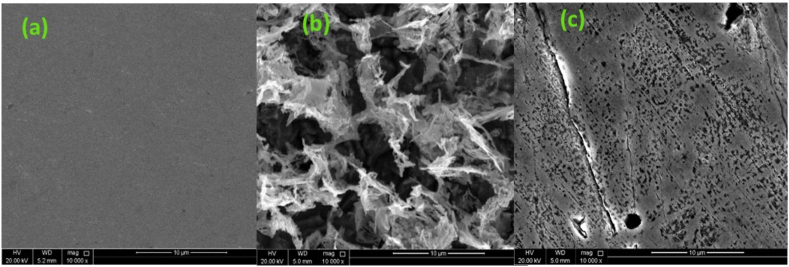
SEM micrographs (a) polished C-steel immersed in 1.0 M HCl (b) in the absence and (c) presence of 50 mg L^−1^ BPUA for 12 h at 25 °C.

SEM was performed to examine the C-steel electrode surface topography before and after the corrosion properties investigation of BPUA in 1.0 M HCl medium. Fig. 5 (a) displays the smooth and uniform C-steel surface before corrosion experiments without defects. Immersion of the C-steel specimen in the uninhibited 1.0 M HCl medium has severely corroded the surface, causing dense cracks with deep holes and pittings, as shown in Fig. 5 (b). The BPUA inhibitor effect was remarkably pronounced in suppressing the corrosion, relying on the SEM micrograph in Fig. 5 (c). The addition of BPUA has significantly reduced the surface roughness, confirming the formation of a dense protective barrier due to the adsorption of inhibitor molecules onto the C-steel surface. Indeed, the morphology analysis proves the findings of electrochemical measurements and asserts the corrosion inhibition capabilities of BPUA.

### 3.5 Eco-toxic evaluation

#### 3.5.1 Experimental approach

**Fig. 6:**
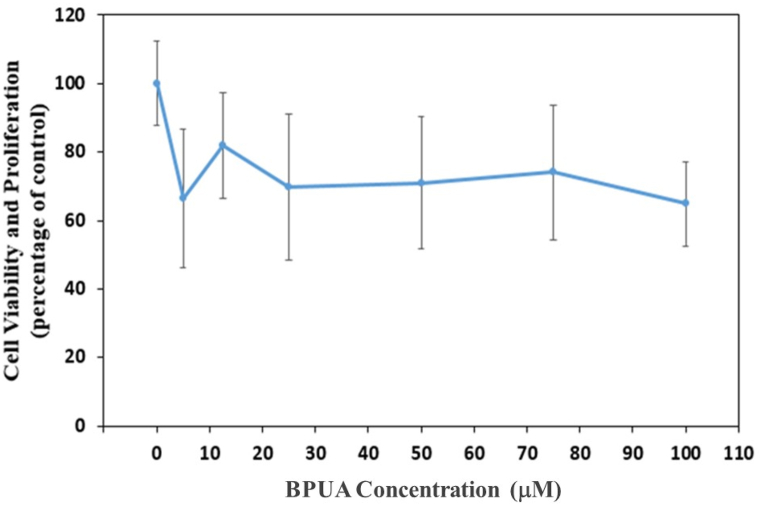
Effect of BPUA concentration on the MCF-10A cell viability and proliferation.

The cytotoxic effect of BPUA on the cell viability and proliferation of human epithelial MCF-10A cells was examined [53,54]. It is obvious that the treatment of the epithelial cells with a wide range of concentrations reaching 100 μM for 24 h caused minimal changes in the cell viability or alterations in its proliferation (Fig. 6). In detail, the exposure of MCF-10A for 15 μM BPUA inhibitor prompted around a 20% reduction of cell viability, reaching 80%. The impact of increased BPUA doses was not remarkably noticed in causing destructive consequences on cell viability. The maximum investigated exposure of 100 μM BPUA inhibitor compound in the MCF-10A cells successfully elucidates sustaining approximately 70% cell viability. Therefore, the toxicity assessment discloses a safe inhibitor compound with negligible detrimental effects on human cells.

### 3.6 Theoretical approach

The probabilities of the fundamental eco-toxicity descriptors for the assessment of BPUA are reported in Table 4. It is evident that the chemical structure of BPUA is in the acceptable domain of applicability, ascribed to the safe predictions of all considered parameters. Obtained data discloses more than 90% of safe probabilities predicted for eye corrosion and irritation, skin sensitization, and honey bee toxicity. Nevertheless, the BPUA structure is recognized as biodegradable with a high prediction of 93%, demonstrating its effortless.

**Table 4:** Probabilities of eco-toxicity evaluation parameters for the BPUA inhibitor.

Applicability domain	In domain
Carcinogenicity	0.79 (safe)
Eye corrosion	0.98 (safe)
Eye irritation	0.93 (safe)
Ames mutagenesis	0.64 (safe)
skin sensitization	0.90 (safe)
Acute oral toxicity	0.71 (slightly toxic)
Honey bee toxicity	0.99 (safe)
Biodegradation	0.93 (safe)
Crustacea aquatic toxicity	0.50 (safe)
Water Solubility (Log S)	−3.72 (soluble)

disintegration by microorganisms with time. Besides, the investigated molecule recorded a 79% prediction for possessing safe and non-carcinogenic impacts. The probabilities decline to 50% and 64% for Ames mutagenesis and crustacean aquatic toxicity, maintaining safe properties. This certainly demonstrates the incapability of BPUA to cause mutations in the DNA of living organisms. Besides, BPUA possesses 71% for slightly toxic impacts when considering acute oral toxicity. The acquired water solubility indicates a soluble nature for BPUA inhibitor, yet this value must exceed zero to comply with the classification of highly soluble compounds [29].

### 3.7 Quantum chemical calculations

**Fig. 7:**
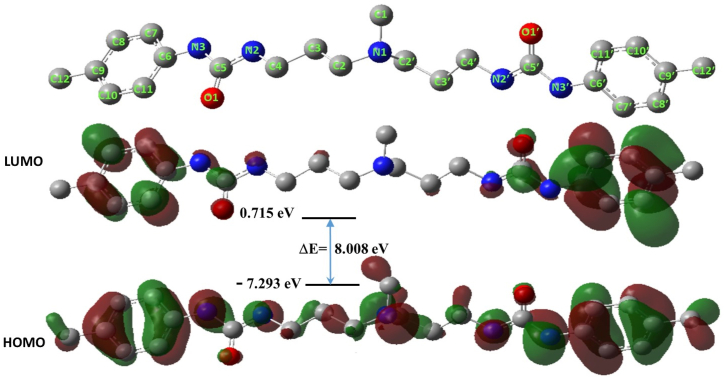
Selected frontier MOs of II and III and the corresponding electronic transitions; DFT (B3LYP/6-31G+(d), IEFPCM, water); hydrogen atoms were omitted for clarity.

As discussed above, the findings of the experiments were employed for modeling inhibitor adsorption. In principle, the difference in the absolute electronegativity between the inhibitor molecules and the metal is a crucial factor for the consequent interaction between them, where the electrons tend to flow along the electronegativity gradient toward establishing an equivalency in chemical potentials. Accordingly, DFT-based and quantum computations were performed to validate the proposed mechanism of adsorption of BPUA on the surface of the C-steel that was presented. In principle, the frontier molecular orbital (FMO) theory of chemical reactivity proposes that the orbital energy gap (ΔE) is a crucial chemical parameter that may be used to characterize various physicochemical properties of materials [[55], [56], [57], [58]]. Accordingly, the adsorption of inhibitor molecules on metallic surfaces can be assessed based on the FMO, where this phenomenon is attributed to the possibility of interaction between the HOMO and LUMO states of reacting species. As such, the strength of the interaction of the adsorbate with the adsorbent is inversely proportional to ΔE. To this end, selected global and local quantum descriptors for the BPUA-C-steel interaction were calculated on the basis of the optimized geometry of BPUA in its neutral, anionic, and cationic forms (Table 5). The optimized structure of the neutral state of BPUA in implicit aqueous solutions and the HOMO/LUMO distributions are displayed in Fig. 7.

**Table 5:** Global quantum parameters of the BPUA.

ΔE eV	I eV	A eV	χ eV	η eV	ΔN
8.008	7.293	−0.715	3.289	4.004	0.463

Examining the optimized geometry, one can notice that the BPUA derivative exhibits roughly planar geometry. Such planarity in the geometry suggests that all functional groups of the molecule are potentially available for donor-acceptor (D-A) electronic interactions with the surface of the metal. Additionally, examining the HOMO of BPUA, it can be noticed that the HOMO is distributed throughout the entire molecule, suggesting multi-cooperative D-A interactions, including π-staking. This is indicative of inhibitor→metal electronic interaction. On the other hand, examining the LUMO, one can notice that it is distributed over the phenyl rings, indicative of a more localized electrophilic character for the inhibitor molecule, indicative of metal→inhibitor electronic interaction. However, per the distribution of the HOMO and LUMO, one may suggest that the inhibitor→metal electronic interaction is more dominant. Nevertheless, it can be suggested that the cooperative inhibitor→ metal and metal→inhibitor electronic donations indicate excellent inhibition behavior for BPUA [59,60]. Additionally, the DFT calculations revealed a value of 0.463 for ΔN, indicating an electronic donation of the type inhibitor→ metal [59,61,62], which is in good agreement with the nature of the HOMO and LUMO distributions.

**Fig. 8:**
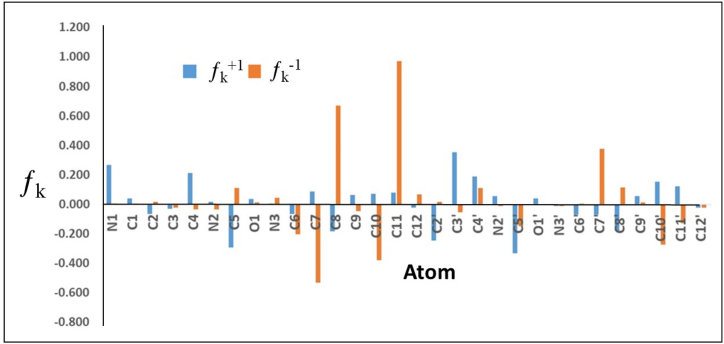
Fukui indices for the BPUA inhibitor molecule; (DFT (B3LYP/6-31G+(d), IEFPCM, water)).

The local quantum descriptor that ascribes the reactivity of inhibitor molecules is presented according to Fukui indices [[63], [64], [65], [66]]. These indices indicate the sites on the molecule that are susceptible to electrophilic (ƒ_k_^−1^) and nucleophilic (ƒ_k_^+1^) attacks, which are indicative of the reactivity of zones present in the inhibitor molecule responsible for establishing potential cooperative interactions with the surface of the metal. In turn, this localized donor–acceptor interaction accounts for the adsorption process of the inhibitor molecules on the surface of the metal. To this end, the ƒ_k_^+1^ and ƒ_k_^−1^ indices of BPUA molecules in their neutral forms were calculated based on optimized geometry of the neutral, cationic, and anionic forms obtained employing the DFT (B3LYP/6-31G+(d), IEFPCM, water). The obtained results are summarized in Table 6, and the relative values of all atoms are displayed in Fig. 8.

**Table 6:** Calculated Fukui indices for the BPUA inhibitor molecule; (DFT (B3LYP/6-31G+(d), IEFPCM, water)).

Atom	Charge	ƒ_k_^+1^	ƒ_k_^−1^
N1	−0.081	0.267	0.005
C1	−0.500	0.042	0.005
C2	−0.569	−0.064	0.019
C3	−0.142	−0.031	−0.021
C4	−0.494	0.215	−0.033
N2	−0.536	0.018	−0.032
C5	0.927	−0.290	0.113
O1	−0.702	0.037	0.014
N3	−0.616	0.005	0.045
C6	−0.002	−0.066	−0.202
C7	−0.951	0.090	−0.532
C8	−0.137	−0.183	0.671
C9	0.379	0.064	−0.045
C10	−1.081	0.071	−0.378
C11	1.046	0.082	0.971
C12	−0.772	−0.021	0.067
C2′	−0.341	−0.243	0.018
C3′	−0.460	0.355	−0.054
C4′	−0.341	0.188	0.110
N2′	−0.576	0.057	−0.010
C5′	0.915	−0.330	−0.148
O1′	−0.701	0.040	−0.001
N3′	−0.600	−0.008	−0.008
C6′	0.053	−0.069	0.004
C7′	−0.779	−0.070	0.379
C8′	−0.063	−0.180	0.114
C9′	0.360	0.058	0.013
C10′	−1.202	0.153	−0.272
C11′	0.947	0.124	−0.129
C12′	−0.772	−0.023	−0.021

For ƒ_k_^−1^, it can be noticed that several functional groups across the BPUA molecules exhibit positive values indicative of being susceptible to electrophilic attacks by the Fe atoms on the surface of C-steel; this includes the heteroatoms-based functional groups as well as the aromatic rings. However, atoms allocated in the phenyl rings, namely C11, C8, C8ʹ, and C7ʹ, exhibited the highest ƒ_k_^−1^ with values of 0.971, 0.671, 0.379, and 0.114, respectively. These ƒ_k_^−1^ values indicate a more favorable inhibitor→metal electronic donation for these sites. These findings are in good agreement with results reported in the literature, where the Fe atoms exhibited more affinity toward the π-electronic cloud of the aromatic rings [67,68]. Such affinity supports the flat orientation of the adsorbed molecules of BPUA on the surface of the C-steel, which in turn is consistent with the optimized geometry of BPUA.

On the other hand, for ƒ_k_^+1^, comparable behavior to ƒ_k_^−1^ can be noted regarding the centers that are susceptible to nucleophilic attacks. The highest ƒ_k_^+1^ are calculated for C3ʹ, N1, C4, C4ʹ, C10ʹ, and C11ʹ, with values of 0.355, 0.267, 0.215, 0.188, 0.153, and 0.124, respectively. However, comparing the values of ƒ_k_^−1^ against ƒ_k_^+1^, one can suggest that the inhibitor→metal electronic donation is more dominant. These results indicate that BPUA is susceptible to chemisorption via cooperative BPUA→Fe and Fe→BPUA electronic donations. Furthermore, an inhibitor molecule bearing heteroatom-based functional groups present in acidic solution is more vulnerable to protonation. As such, the chemical structure of BPUA features heteroatom-based functional groups, namely amines and carbonyls, which are more susceptible to protonation in 1.0 M HCl solution. Hence, this suggests that physisorption is to be considered as a co-factor that supports the adsorption of BPUA on the surface of C-steel via electrostatic interaction.

### 3.8 Corrosion inhibition mechanism of BPUA

Typically, the two main factors that influence the protection mechanism of inhibitors are the chemical structure and the metallic surface type and charge. The corrosion prevention mechanism is formed by the interaction between the active sites of BPUA and the charged surface of the steel, as shown by the aforementioned experimental and theoretical studies. The immersed C-steel in 1.0 M HCl medium would acquire a negatively charged layer from Cl^−^ adsorption [69]. Amines and carbonyl functional groups of BPUA are highly susceptible to protonation in the HCl solution. This suggests the attraction of positively charged constituents of BPUA towards the negatively charged steel due to the electrostatic force. The BPUA starts repelling the previously adsorbed corrosive species, consequently settling on the surface and shielding the anodic and cathodic reactive sites [70]. At this point, the corrosion mechanism is dominated by the physisorption process. Nevertheless, several heteroatoms in the functional groups and the aromatic rings are subjected to electrophilic attacks by Fe atoms, resulting in BPUA → Fe electronic donations.

**Fig. 9:**
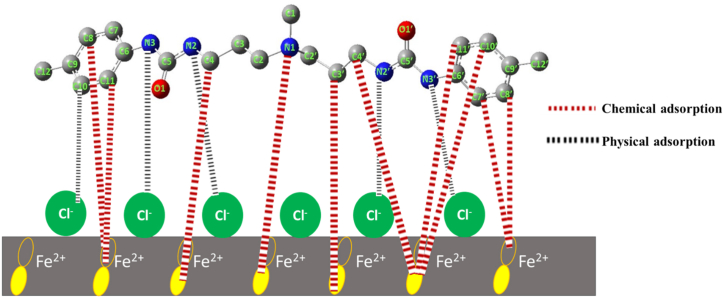
Corrosion mechanism and possible interactions between BPUA inhibitor and C-steel surface.

In contrast, few other atoms show their vulnerability to nucleophilic attacks, suggesting Fe → BPUA electronic donations. Therefore, chemisorption via charge donation between the active sites occurs and blocks the corrosion process. It means the loosely bounded electrons transfer to the unoccupied d-orbitals of Fe atoms [71]. Overall, the distribution of the reactive atoms across the whole BPUA chain induces the flat orientation of adsorbed inhibitor molecules over a wider steel surface to block major active sites (Fig. 9). It is worth mentioning that the molecular weight and large size of BPUA molecules are co-factors supporting its adsorption onto the C-steel surface.

### 3.9 Comparison with other phenyl urea-based inhibitors

Urea-derived organic moleucles have continuously attracted research interests due to their effieicent corrosion inhibition activities. However, the literature studies testing a compound with phenyl and urea groups similar to the developed structure in this paper are rare. Table 7 reports the performance of a few similar structures based on phenyl-urea groups in the acidic medium under different conditions and steel types. A comparative analysis of the results obtained confirms the superior behavior of the synthesized BPUA compound to inhibit corrosion and achieve 95% efficiency.

**Table 7:** Performance of phenyl-urea based inhibitors reported in the literature.

Compound	Medium	C_Inh._ (mg.L^−1^)	IE%	Ref.
1-(4-Aminophenyl)-3-(adamantan-1-yl) urea(1-4-APADU)	Mild steel corrosion in 1.0 M HCl at 25 °C	20 60	60.5 79.1	[72]
1-(4-Aminophenyl)-3-octylurea (1-4-APOU)		20 60	87.2 91.0	
1-(4-Aminophenyl)-3-dodecylurea(1-4-APDDU)		20 60	89.1 90.6	
1-(4-Aminophenyl)-3-octadecylurea(1-4-AP ODU)		20 60	49.5 65.7	
1,3-Bis(1-phenylethyl) urea	Carbon steel corrosion in 1.0 M HCl at 32 °C	20 60	31.6 50.5	[21]
1,3-Bis-[phenyl-(pyridin-2-yl amino)-methyl]-urea (pabu)	Mild steel corrosion in 10% HCl at 30 °C	150	33.6	[73]

## 4 Conclusions

In the present study, as a potential green inhibitor, a novel derivative of bis-phenylurea-based aliphatic amine (BPUA), namely 1,1'-((methylazanediyl)bis(propane-3,1-diyl))bis(3-(*p*-tolyl)urea), exhibited excellent inhibitory behavior against the corrosion of C-steel in 1.0 M HCl medium. The BPUA compound was synthesized employing a facile synthetic route, and spectral characterization using NMR spectroscopic experiments proved the suggested structure. The effectiveness of the BPUA as a corrosion inhibitor is demonstrated by the results obtained by the electrochemical EIS and Tafel measurements. Corrosion protection efficiency of 95.1% was achieved for C-steel in the studied corrosive medium, where the presence of 50 mg L^−1^ BPUA substantially increased the charge transfer resistance from 196 to 4040 Ω cm^2^. Over the studied rane of concentrations, the BPUA inhibitor displayed an excellent performance in reducing the corrosion rate by around 19 times, from 33 to 1.7 mils per year. In addition to that, the analysis of adsorption isotherms determined a Gibb's free energy between −35 and −37 kJ mol^−1^. This indicates a spontaneous corrosion inhibitory action of BPUA with physisorption/chemisorption modes obeying Langmuir isotherm. Eco-toxicity assessment of BPUA structure using ADMET prediction software supported the environmental-friendliness properties of BPUA with negligible harmful impacts. Furthermore, the cytotoxic effect against the human epithelial cells MCF-10A disclosed a successful cell viability preservation of around 70% at an exposure limit of 100 μM BPUA. DFT-based quantum chemical calculations supported the experimental findings. Acquired results by the selected global and local quantum descriptors, including the FMO theory and Fukui indices, depicted that the BPUA inhibitor can be adsorbed on the surface of C-steel via flat orientation with strong interaction that comprises the heteroatom-based functional group as well as the aromatic rings of the inhibitor molecules.

## Author contribution statement

Mohanad Shkoor; Rem Jalab; Tahseen S. Shawkat: Performed the experiments.

Mazen Khaled: Conceived and designed the experiments; Analyzed and interpreted the data; Wrote the paper.

Hesham M. Korashy: Analyzed and interpreted the data.

Mohamed Saad: Analyzed and interpreted the data; Contributed reagents, materials, analysis tools or data.

Haw-Lih Su: Contributed reagents, materials, analysis tools or data.

Abdulilah Dawoud Bani-Yaseen: Conceived and designed the experiments; Performed the experiments; Analyzed and interpreted the data; Wrote the paper.

## Data availability statement

Data will be made available on request.

## Declaration of competing interest

The authors declare that they have no known competing financial interests or personal relationships that could have appeared to influence the work reported in this paper.
