# Supplementary material for: Experimental and theoretical investigations of the effect of bis-phenylurea-based aliphatic amine derivative as an efficient green corrosion inhibitor for carbon steel in HCl solution
Source: Heliyon. 2023 Sep 22;9(10):e20254. doi: 10.1016/j.heliyon.2023.e20254 (PMC10539979; doi:10.1016/j.heliyon.2023.e20254)
Supplement: Multimedia component 1 [file mmc1.docx]

**A Novel Bis-phenylurea-Based Aliphatic Amine as an Efficient Green Corrosion Inhibitor for Carbon Steel in HCl Solution: Experimental and Theoretical Study**

**EXPERIMENTAL**

**General**

All reagents including the salicylaldehydes, ethyl 3-aminocrotonate and solvents and were purchased from Sigma-Aldrich and VWR international and were used as received without additional purification. Reactions monitored by thin-layer chromatography (TLC) on silica gel 60 F254 using UV light.

^1^H and ^13^C spectra were recorded on a JOEL 600 MHz spectrometer. ^1^H NMR spectra were internally referenced to the residual solvent signal (CHCl_3_ = 7.24 ppm). ^13^C NMR spectra were internally referenced to the solvent signal (CDCl_3_ = 77.00 ppm).

Melting points (^0^C) were performed on open capillaries using an electrothermal digital melting point apparatus and were uncorrected.

Mass spectra were recorded on Agilent 7890B GC/ 5975C MS.


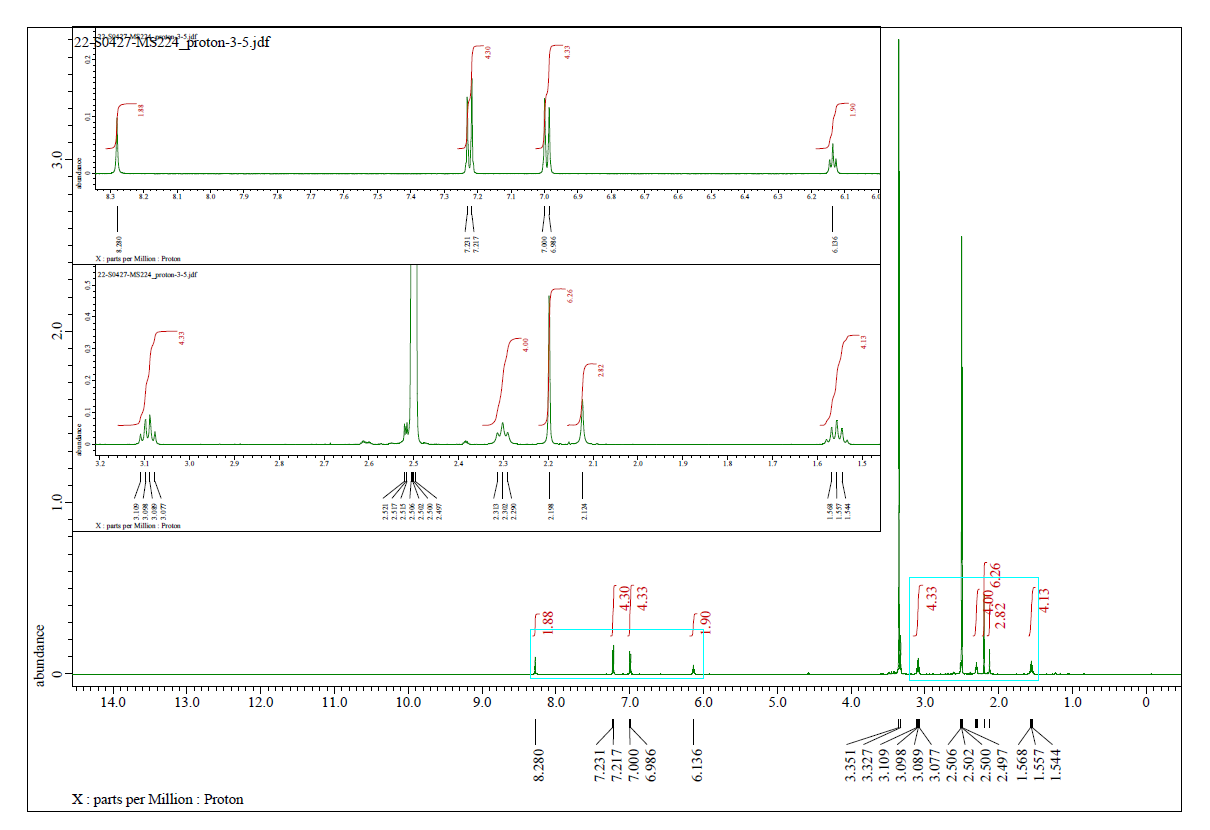


Figure S-1: ^1^HNMR spectrum of compound **BPUA**


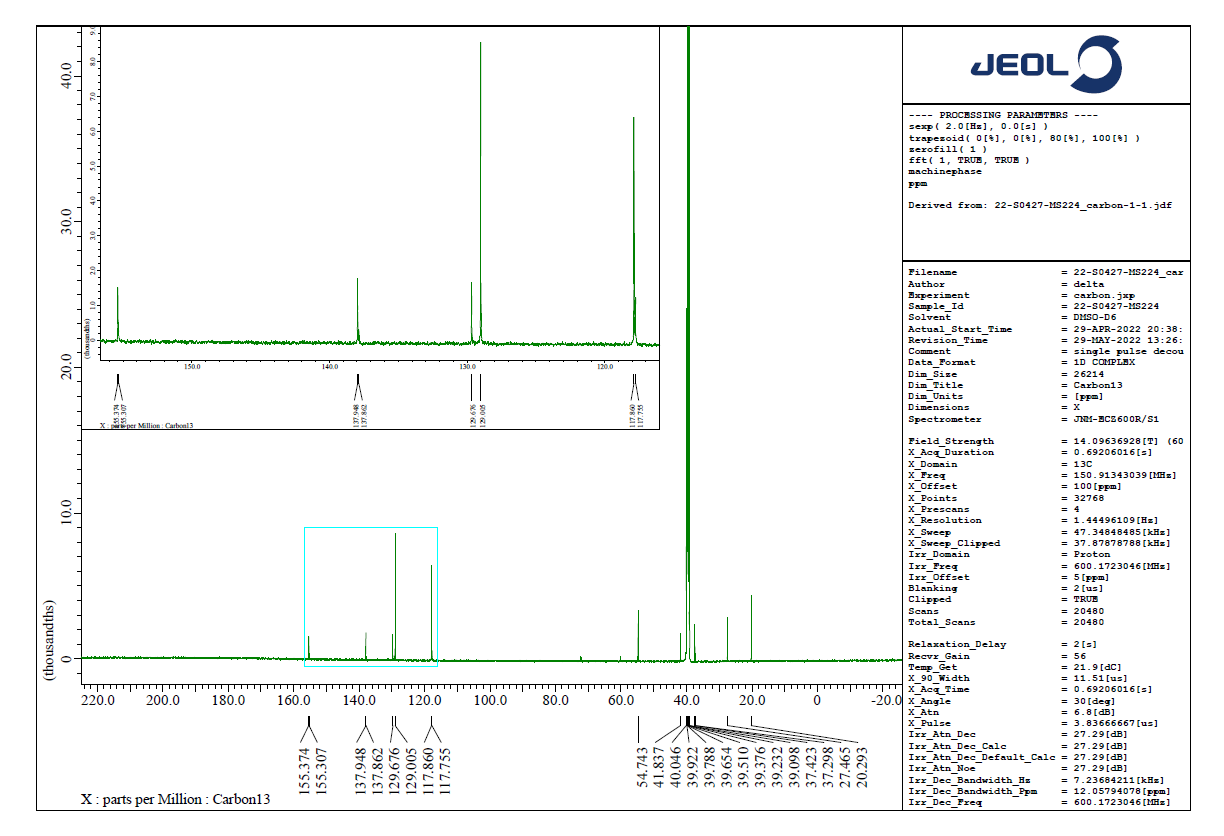


Figure S-2: ^13^C-NMR spectrum of compound **BPUA**


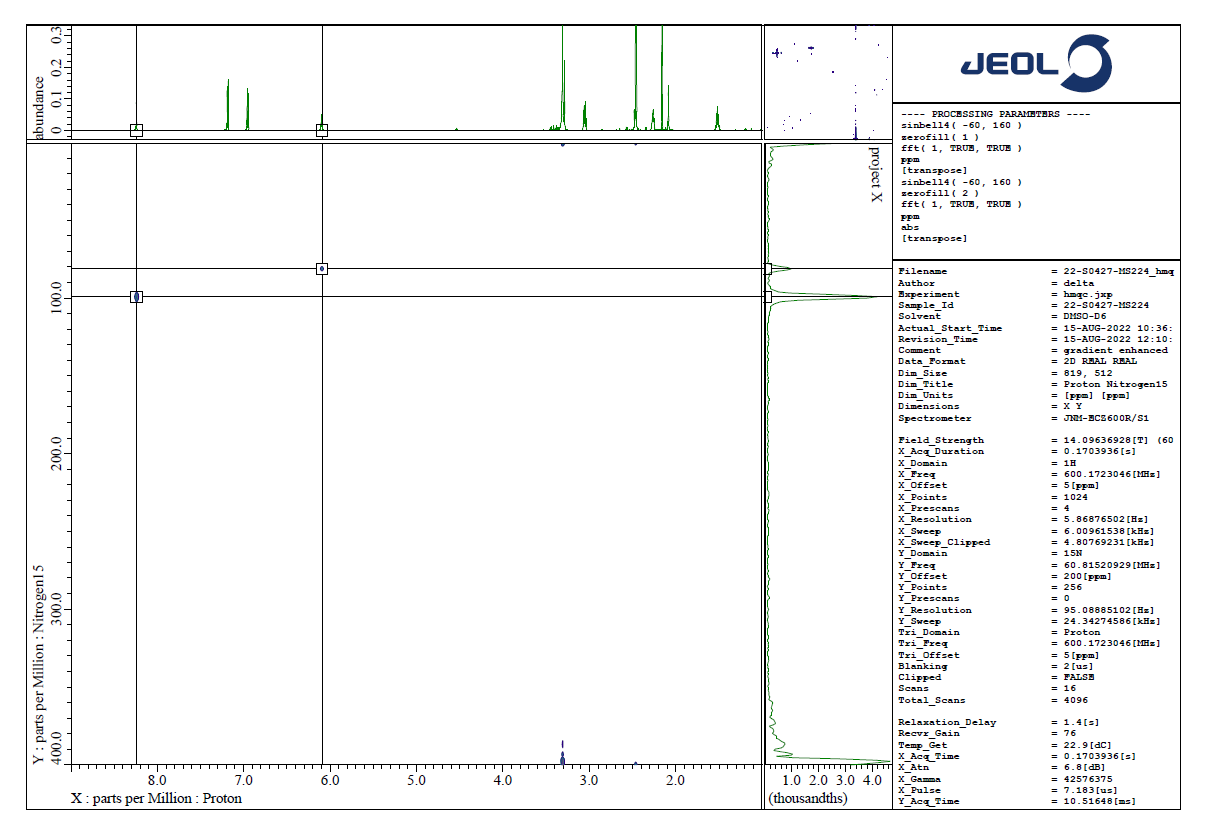


Figure S-3: ^1^H-^15^N HMQC NMR spectrum of compound **BPUA**


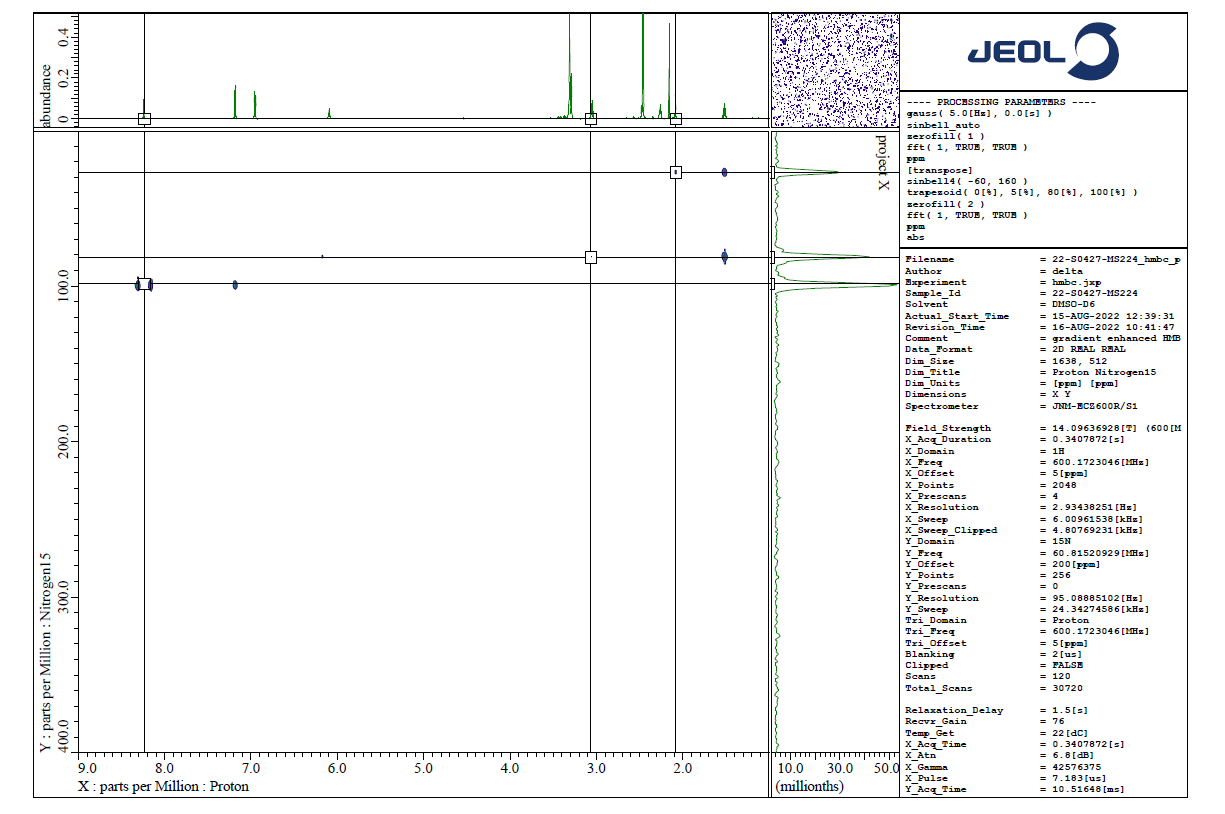


Figure S-4: ^1^H-^15^N HMBC NMR spectrum of compound **BPUA**


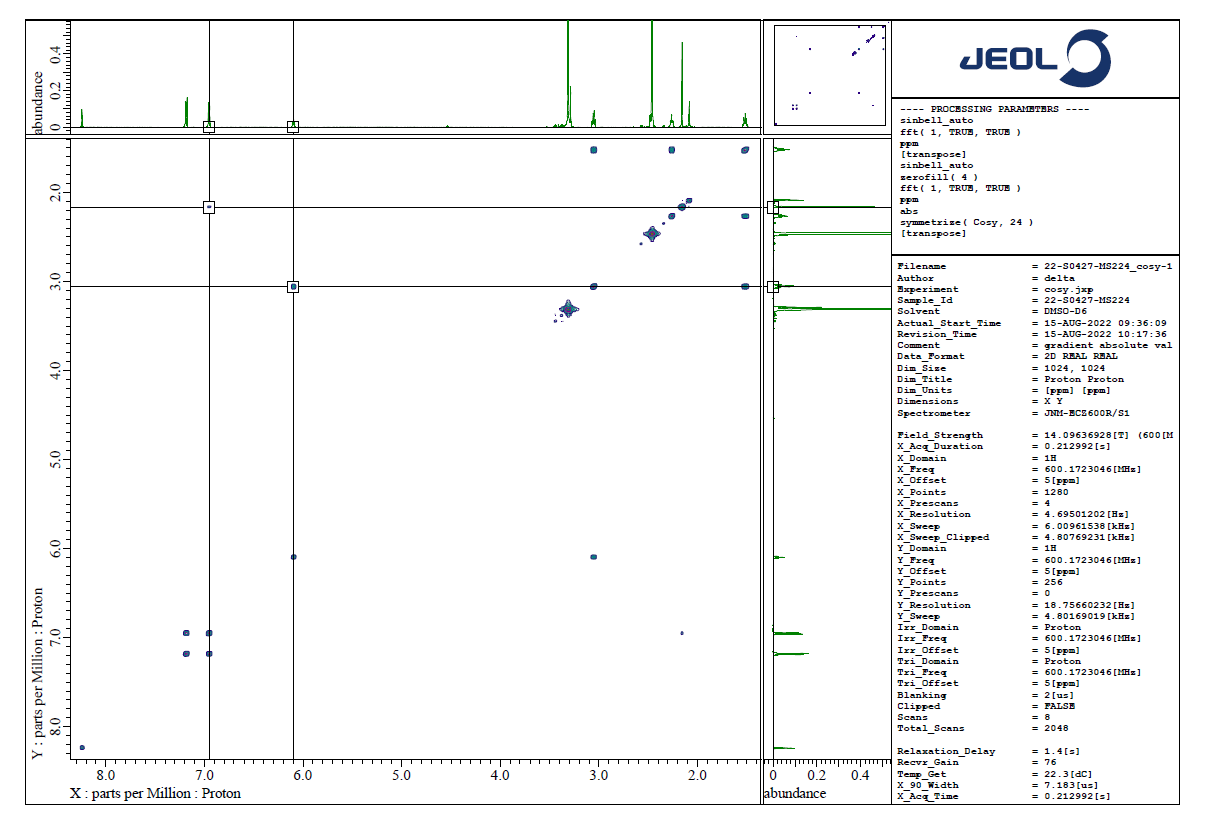
 Figure S-5: ^1^H-^1^H COSY-NMR spectrum of compound **BPUA**


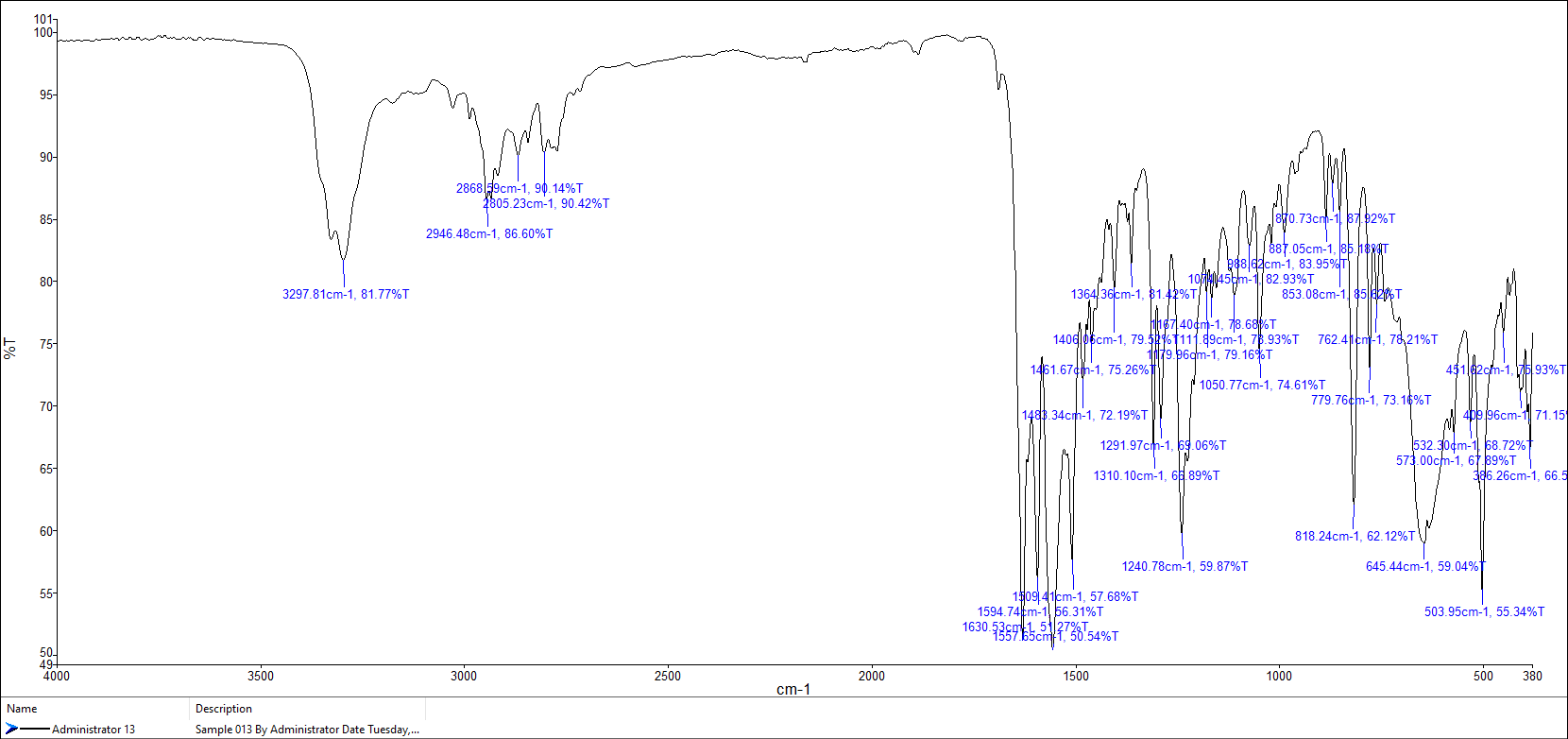


Figure S-6: IR spectrum of compound **BPUA**


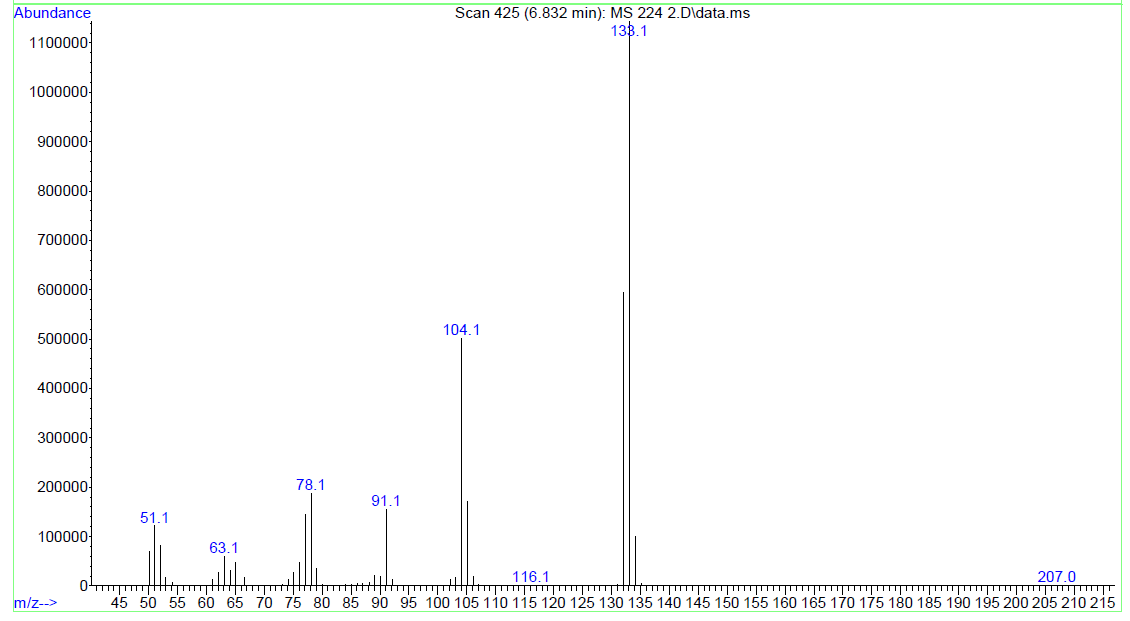


Figure S-7: GC-MS Spectrum of compound **BPUA**
